# Primary and Secondary Organic Aerosol Formation from Asphalt Pavements

**DOI:** 10.1021/acs.est.3c06037

**Published:** 2023-11-06

**Authors:** Mackenzie B. Humes, Jo E. Machesky, Sunhye Kim, Oladayo J. Oladeji, Drew R. Gentner, Neil M. Donahue, Albert A. Presto

**Affiliations:** † Department of Chemical Engineering, Carnegie Mellon University, Pittsburgh, Pennsylvania 15213, United States; ‡ Department of Chemical & Environmental Engineering, Yale University, New Haven, Connecticut 06511, United States; § Department of Mechanical Engineering, Carnegie Mellon University, Pittsburgh, Pennsylvania 15213, United States; ∥ Department of Chemistry, Carnegie Mellon University, Pittsburgh, Pennsylvania 15213, United States

**Keywords:** asphalt, secondary organic aerosol, polycyclic aromatic hydrocarbon, activity factor, semi-volatile organic compound

## Abstract

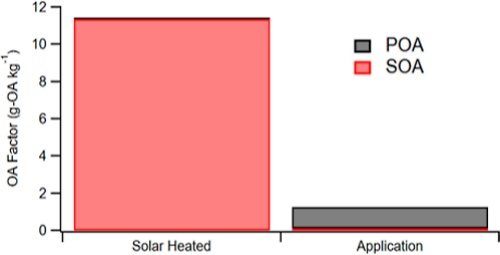

Asphalt is ubiquitous across cities and a source of organic compounds spanning a wide range of volatility and may be an overlooked source of urban organic aerosols. The emission rate and composition depend strongly on temperature, but emissions have been observed at both application temperatures and surface temperatures during warm sunny days. Here we report primary organic aerosol (POA) emissions and secondary organic aerosol (SOA) production from asphalt. We reheated real-world asphalt samples to application-relevant temperatures (∼130 °C) and typical summertime road-surface temperatures (∼55 °C) and then flushed the emitted vapors into an environmental oxidation chamber containing ammonium sulfate seed particles. SOA was then formed following the photo-oxidation of emissions under high-NO_*x*_ conditions typical of urban atmospheres. We find that POA only forms at application temperature as it does not require further oxidation, whereas SOA forms under both conditions; with the resulting POA and SOA both being semi-volatile. While total OA formation rates were substantially greater under the limited time spent under application conditions, SOA formation from passive asphalt heating presents a potential long-term source, as heating continues for the lifetime of the road surface. This suggests that persistent asphalt solar heating is likely a considerable and continued source of summertime SOA in urban environments.

## Abstract 2

This work demonstrates asphalt forms substantial organic aerosol following heating and atmospheric oxidation, and real-life asphalt pavements may be a significant secondary organic aerosol contributor.

## 1 Introduction

Research on the formation of anthropogenic primary and secondary organic aerosols (P/SOA) has for the most part focused on “traditional” combustion sources such as mobile emissions from gasoline and diesel exhaust.^1^ However, the contribution to SOA from these traditional sources has been decreasing over time due to a combination of factors including, but not limited to, catalytic converters, vehicle regulations, gasoline and diesel standards, and increased engine efficiency.^1−3^ Non-traditional sources, by contrast, have gained importance as contributors of SOA precursors due in part to the decline of emissions from traditional sources and include a wide range of source types, including personal care products, paints, cleaning solvents, and inks.^1,4,5^ SOA research has increasingly focused on volatile chemical products, but there may be other unrecognized yet important non-traditional SOA sources.

One potential understudied SOA source is the photo-oxidation of emissions from asphalt pavements. Asphalt is ubiquitous in urban environments throughout the world and has the potential to generate OA through direct POA emission from heating and SOA formation from the gas-phase oxidation of asphalt vapors emitted during application or with increased off-gassing emissions on warm, sunny days.

The potential of asphalt emissions as a source of organic aerosols is derived from the asphalt’s complex chemical structure. Paving asphalt consists of a mixture of aggregates with a complex petroleum binder containing hundreds of species of alkanes, naphthenic hydrocarbons, polycyclic aromatic hydrocarbons (PAHs), and heterocyclic compounds, which can act as a source of organic compounds spanning a wide range of volatility, from volatile organic compounds (VOCs) to low-volatility species, including intermediate volatility and semi-volatile organic compounds (IVOCs, SVOCs).^6,7^ Many of these compounds and their oxidation products are traditionally associated with mobile and industrial sources, so accounting for SOA formation from asphalt may explain a fraction of the under-prediction in current SOA models.^8,9^ OA from asphalt could further resemble established OA factors such as hydrocarbon-like OA (HOA) and oxygenated OA (OOA) observed in ambient data sets. Khare et al. also estimated that asphalt-related materials are a notable missing urban source of intermediate and semi-volatile organic compounds in southern California; however, additional studies are necessary to further constrain their contribution to urban particulate matter concentrations.^7^

Real-world asphalt emissions may occur at various temperature regimes, including higher temperatures during the initial application of asphalt and lower temperatures with passive heating of asphalt during hot summer days. The emission rate and composition depend strongly on the temperature, making the former a greater potential source of shorter-term asphalt emissions. For example, Khare et al. showed that emission rates are significantly higher at application temperatures (∼130–160 °C) than summertime road surface temperatures (∼50–70 °C), and that the composition of the emissions depends on factors such as the presence of UV radiation.^7^ However, these enhanced emissions from paving occur only when new asphalt is paved, and the surface subsequently spends a much longer time, often years, under conditions where passive heating emissions can occur.

During and immediately following application, asphalt produces aerosol mass through the release and subsequent condensation of low-volatility gas-phase organic species.^6^ Evaporation and subsequent recondensation of semi-volatile and low-volatility organic compounds (SVOCs and LVOCs) at these elevated temperatures have sometimes led to particle concentrations of 100+ μg/m^3^ near areas of active road paving.^10^

The composition of SOA from asphalt is poorly understood, and the emission rates of SOA precursors from asphalt are lacking from OA inventories, especially for long-term emissions.^7^ Recent laboratory work from Kriech et al. and Lasne et al. demonstrated that emissions of gas-phase organic compounds from asphalt continue over 17 years after paving.^11,12^ As asphalt pavements remain in place for years, if not decades, before repaving, continuous emissions from already paved asphalt surfaces could be a substantial ongoing source of SOA precursors in urban environments, potentially surpassing initial application. This is likely most significant on hot, sunny days, when the dark asphalt surface can reach temperatures well above ambient air.

Quantifying such long-term emissions is especially important with regard to air quality. In contrast to sources such as vehicle exhaust, asphalt is a stationary yet distributed source and a continuously used product. Hence, any emissions from asphalt would be difficult to control beyond the initial formulation and paving. Understanding the SOA production from asphalt emissions would not only help to account for a missing source of SOA but also provide a lower bound for urban background SOA.

## 2 Materials and Methods

For all experiments, we heated asphalt samples, passed the emissions into a 10 m^3^ Teflon chamber, and photo-oxidized them under high NO_*x*_ conditions via HONO photolysis. Prior to asphalt heating, we generated ammonium sulfate particles by nebulizing a dilute solution in water (2.5 g/L, >99% Sigma-Aldrich, St. Louis, MO). These ammonium sulfate seed particles served as a condensation surface for both primary asphalt emissions and the subsequent SOA formed during photo-oxidation. We injected deuterated butanol (*d*_9_-butanol; 98% Cambridge Isotope Laboratories) into the chamber through a heated septum injector to act as an OH tracer based on a reaction rate coefficient with OH of *k* = (3.4 ± 0.88) × 10^–12^ cm^3^ molecule^–1^ s^–1^.^13^

Asphalt heating emissions were injected into the chamber by placing asphalt samples inside of a 1 in. (2.56 cm) outside diameter heated metal tube. The tube temperature was controlled by using a thermocouple placed on the outside surface of the metal tube. We used two temperature set points: 160 °C (∼130 °C inside the tube after accounting for losses and heat transfer) was used to mimic application temperature and 70 °C (∼55 °C inside the tube) to represent elevated summer daytime road surface conditions. Emissions were transferred into the chamber using a flow of 1 slpm air. Asphalt heating took place in the absence of UV lights, which have been demonstrated to increase emissions.^7^ Hence, the emissions we measured without the presence of UV light may be treated as conservative, lower-bound estimates.

Application temperature experiments used approximately 40 g of asphalt (binder and aggregate) and were heated for 1–1.5 h. Summertime temperature experiments used ∼120 g and were heated for 3 h, which was higher to account for roughly 3 times lower emission factors compared to application temperature conditions under similar loadings.^7^ Measurements of VOCs with a proton transfer reaction mass spectrometer (PTR-MS) were used to check whether our different loadings led to similar emission rates for the two temperature regimes. VOC emissions rates of 0.34 and 0.32 μg m^–3^ min^–1^ were measured for application and summertime temperature, respectively, indicating that our procedures produced similar VOC mass emission rates for each temperature, though there are expected differences for IVOC/SVOCs.

Asphalt pavement samples were collected on May 31, 2022, shortly after paving and cooling from Beelermont Pl., a small road in eastern Pittsburgh, PA, and are independent of asphalt samples used in prior work.^7^ The asphalt samples were sealed tightly and stored in a chemical freezer to prevent vapor off-gassing. For experiments, a sample was removed from the freezer and allowed to reach room temperature, but it remained sealed until weighing. Hence, the asphalt used in this study was heated to application temperatures and used in paving prior to the experiment. As a result, our measurements for application temperature experiments may act as a lower bound for emission estimates.

To produce HONO, we created 25 mL of dilute sodium nitrite (10 g/L, > 99% Sigma-Aldrich, St. Louis, MO) solution and reacted it with near-pure sulfuric acid (95/98% Sigma-Aldrich, St. Louis, MO). We bubbled the product into the chamber and used UV lights (peak λ = 368 nm) to photolyze HONO and form OH under high NO_*x*_ conditions. Experiments in the chamber took place at a NO_*x*_ concentration of 2.5 ppm, an OH concentration of ∼1 × 10^7^ molecules cm^–3^, low (<10%) relative humidity, and a temperature of 25 °C.

Gas phase species, including the d-9 butanol tracer, were measured using a quadrupole proton transfer reaction mass spectrometer (PTR-MS, Ionicon Analytik, Innsbruck, Austria). The PTR-MS was calibrated using a standard calibration gas mixture (Airgas Specialty Gases, Plumsteadville, PA), as shown in Table S1 in the Supporting Information. For particle phase measurements, we used an aerosol mass spectrometer (AMS) to determine the particle mass concentration and composition. The AMS is a hard-ionization instrument employing electron ionization (EI), leading to a high fragmentation. Hence, particle-phase mass spectra from the AMS are usually associated with characteristic fragments rather than individual species. However, aromatic species are more robust against EI fragmentation and so the parent ions can sometimes be identified in AMS spectra.^14^ AMS data were analyzed using Squirrel (version 1.61) and PIKA (version 1.21) in Igor (v. 7.08).

## 3 Results and Discussion

### 3.1 Organic Aerosol Production

**Figure 1:**
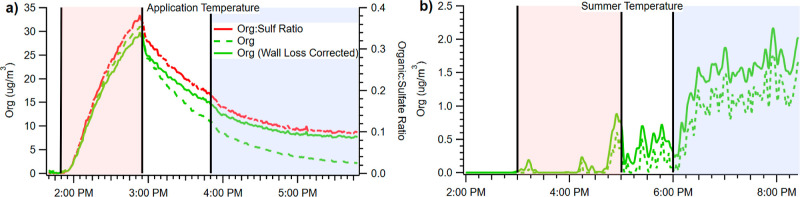
Concentration of organic aerosol vs time for heated asphalt at application temperature (a) and at summer temperature (b). At summer temperature, no significant POA production occurred, whereas photo-oxidation (blue shading) formed SOA. At application temperature, substantial POA was produced, followed by wall loss of organics, which was then countered by SOA formation. The decrease in the organic/sulfate ratio after heating indicates the loss of organic vapors to the walls and is consistent with semi-volatile constituents.

Heating asphalt to the application temperature (red-shaded region in Figure 1a) produced POA (∼30 μg/m^3^). After sample injection, the OA concentration declined due to both particle wall loss and the uptake of organic vapors to the chamber walls, which caused the ratio of OA to the seed mass to steadily drop, as shown in Figure 1a.^15,16^ During photo-oxidation (blue-shaded region in Figure 1a), the loss of POA to the chamber wall was countered by substantial SOA formation. Hence, the wall loss-corrected OA concentrations eventually stabilized, with wall losses approximately balancing continued SOA formation. Determining the mass of SOA formed necessitates separating POA already present and accounting for their wall losses.

Asphalt heated to summer temperature conditions, as shown in Figure 1b, did not produce significant POA despite the presence of ammonium sulfate seeds. By contrast, after high NO_*x*_ photo-oxidation, SOA did form. Within 1 h, the wall loss-corrected SOA reached a plateau near 1.5 μg/m^3^. Therefore, even though OA is produced more readily at application temperature, at summer temperature conditions SOA is still able to form, suggesting that post-application asphalt still has considerable SOA potential.

### 3.2 Wall Loss and Volatility

**Figure 2:**
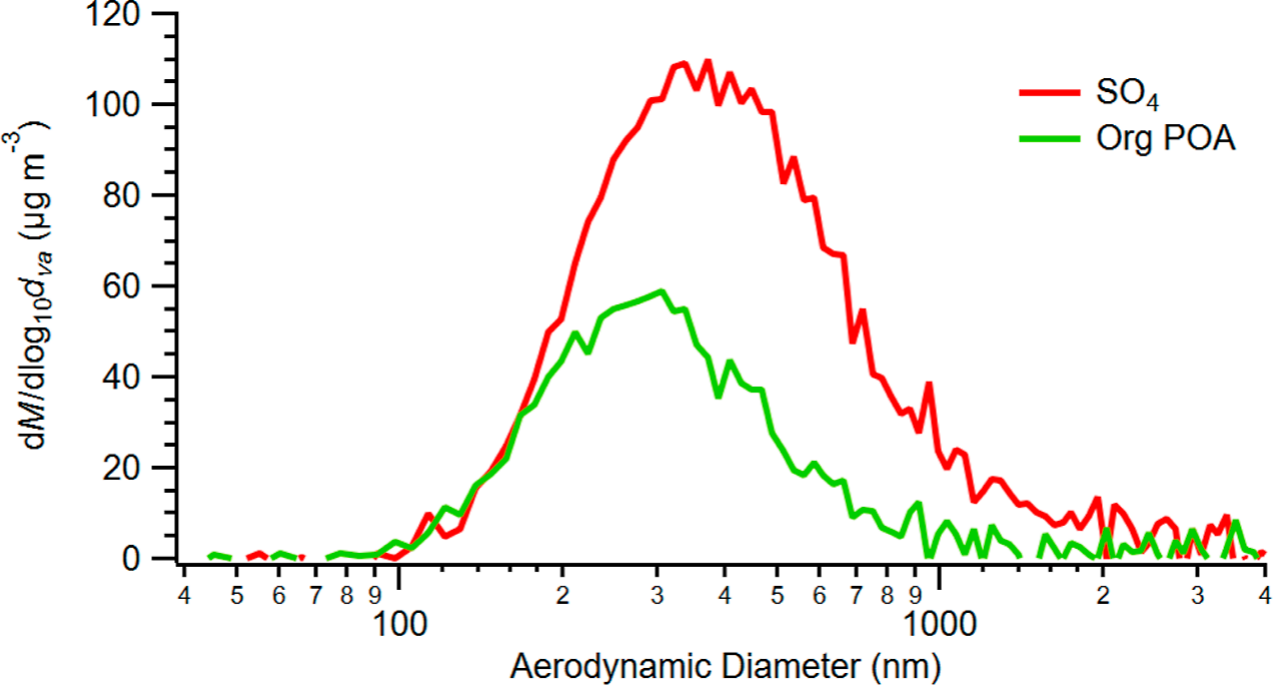
Semilog plot of mass concentration versus diameter for POA from application temperature asphalt along with sulfate from seed particles. The distributions for both organic constituents and seeds are generally singular log–normal peaks centered at roughly 300 and 400 nm, respectively. The organic constituents falling almost entirely on the seeds and the lack of a second log–normal curve centered in the 10–100 nm diameter size indicate that condensation dominates over nucleation.

A major confounding factor in all Teflon chamber experiments is the particle and vapor losses to the wall. The ammonium sulfate seeds serve two roles: they minimize (or at least buffer) vapor losses to walls by acting as a condensation sink for organic vapors, and they enable direct quantification of particle wall loss.^17^ Particle wall loss can be corrected for by multiplying the sulfate concentration by the AMS-derived organic/sulfate ratio.^17,18^ This wall loss correction is based on two assumptions: (1) the ammonium sulfate seeds have no further sources after their introduction and (2) the ammonium sulfate and organics have similar wall loss rate constants.^17^ The former holds as we control the introduction of ammonium sulfate seeds, and the latter holds due to overlapping size distributions and mostly large (*D*_p_ > 200 nm) particle formation (Figure 2).^17^ Vapor wall loss, by contrast, can be estimated from the loss of condensed organics from the suspended seed particles when there are no sources of organics (i.e., no emissions or photochemistry).^16^

Organic vapor interactions with Teflon can be modeled via semivolatile partitioning, but for low OA and (relatively) low volatility vapors. Matsunaga and Ziemann^19^ tested the remaining vapor in the chamber after uptake to the chamber walls for a wide range of IVOCs. Consistent with their findings, Ye et al. showed that losses of SVOC vapors are quasi-irreversible.^16,19^ Considering vapor loss to the Teflon walls as quasi-irreversible, we can assume a steady state for the organic vapors and therefore a mass balance between the loss of organic vapors from the seed particles and to the Teflon chamber walls.^16^ From the mass balance, we find the loss rate of organic vapor to the walls is , where *C*_org_^P^/*C*_sulf_^P^ is the measured organic/sulfate ratio, C_sulf_^P^ is the concentration of sulfate, and CE is the collection efficiency of the AMS, which we can estimate at ∼0.2 for ammonium sulfate seed particles.^16,20^ Using this equation, the loss rate can be found in three steps: adding the change in organic/sulfate multiplied by sulfate concentration for each measurement interval (∼1 min) to obtain the total mass loss of organics, graphing this versus time, then performing a linear fit and measuring the slope.^16^

We measured organic/sulfate over time. At application temperature, it rose during asphalt vapor injection, peaked at ∼0.4, and then dropped at a steady rate after injection. During high NO_*x*_ oxidation, the organic/sulfate ratio decreased at a slower rate before stabilizing at ∼0.1 due to SOA formation balancing vapor loss. The organic/sulfate ratio initially declines due to the loss of organic species to the walls after heating is finished. This is then countered by SOA formation increasing the mass of organics on the seeds until an equilibrium is reached, wherein gains from SOA formation counter the loss of organics to the wall.

As a point of comparison, we can observe the change in slope of org/sulfate during oxidation, as highlighted in Figure 1a. During the dark period after asphalt heating and before oxidation, the org/sulfate ratio decreases at a rate of −0.0027 min^–1^. By contrast, during oxidation, the change in the org/sulfate ratio approaches zero over time, from −0.0011 min^–1^ shortly after the start of oxidation at 4:00 PM to −7.0 × 10^–4^ min^–1^ at 4:30 PM and zero by 5:30 PM.

The saturation concentration c* can also be estimated from the organic vapor wall loss rate via the equation: , where *a*_org_^P^ is the condensed phase activity (equal to 1 for pure organics), CS^P^ is the condensation sink to the particles, and CS^W^ is the condensation sink to the walls.^16^ Since CS^P^ is ∼15–30 times greater than CS^W^, the saturation concentration can be estimated based on the wall loss rate and condensation sink to the chamber walls.^16^ The condensation sink to the chamber walls has been estimated to be ∼0.065 min^–1^, meaning that by dividing the vapor wall loss rate by the condensation sink to the chamber walls, we can determine the mean saturation concentrations.^16^ As demonstrated in Section S4 in the Supporting Information, we can use this equation to determine the mean saturation concentrations of the particulate phase of c* = 16 μg m^–3^ for application temperature POA, c* = 6 μg m^–3^ for application temperature post-oxidation, and c* = 2 μg m^–3^ for SOA under the summer temperature regime, with all falling in the SVOC range.^16^

In addition, we measured the mass size distribution of the ammonium sulfate seeds and OA using the particle time-of-flight (PToF) mode of the AMS, shown for the application temperature POA in Figure 2. The organic aerosol and seeds both formed a log–normal size distribution, with the organic mode at a smaller diameter than the seeds. The single organic mode entirely overlapping the seed mode is typical of vapors condensing onto seeds, where the organics are displaced toward lower diameters due to condensation favoring their higher surface area to volume ratios.^21^ Organic aerosols often form a second log–normal mode in the 10–100 nm range via the nucleation of lower volatility species, such as those in the ELVOC and LVOC ranges. The lack of a prominent second mode below 100 nm indicates that the organics condensed onto the seeds rather than nucleating. This demonstrates that asphalt heating in our experiments did not volatilize ELVOCs and LVOCs to any significant extent while still emitting species with sufficiently low volatility to condense onto the seeds. Together with the steady decrease in the organic/sulfate ratio and calculated saturation concentrations, this single, log–normal condensation mode suggests a substantial amount of the asphalt heating emissions were semi-volatile (i.e., SVOCs).

### 3.3 Organic Aerosol Composition

**Figure 3:**
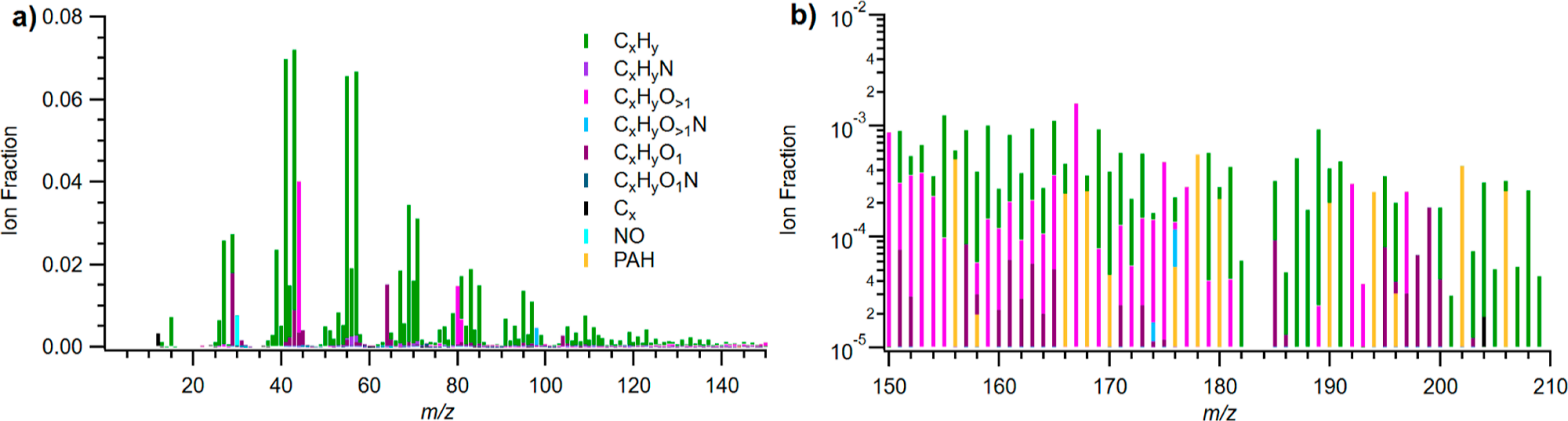
Normalized POA mass spectrum (fraction of signal normalized for all ions) with a linear *y*-axis for *m*/*z* < 150 (a) and a log *y*-axis for *m*/*z* > 150 (b) from application temperature asphalt heating. The fragments are colored based on chemical “families”, where purple indicates oxygenated species, blue represents nitrogenated species, and green represents species without nitrogen or oxygen groups. PAHs are colored orange and visible in the log plot. Overall, non-oxygenated and non-nitrogenated species dominate POA constituents, and PAHs constitute a substantial fraction of the signal at higher *m*/*z*.

Figure 3 shows the composition of the POA at application temperatures, as measured with the AMS normalized for the signal of all ions. The POA is dominated by C_*x*_H_*y*_ ions. These constitute approximately 78% of the POA mass compared with 18% for oxygenated hydrocarbons and 0.9% for N-containing ions. These C_*x*_H_*y*_ groups demonstrate a “picket fence” pattern every 14 *m*/*z*, consistent with adding another carbon to the carbon backbone. Oxygenated groups, by contrast, are spread throughout the mass range, with the largest peak at *m*/*z* 44 corresponding to CO_2_^+^, which consisted of 4% of the total signal. This specific fragment also indicates the extent of oxygenation when compared to the fraction at *m*/*z* 43, which is a combination of C_2_H_3_O^+^ and C_3_H_7_^+^, representing less complete oxygenation.^22^

**Figure 4:**
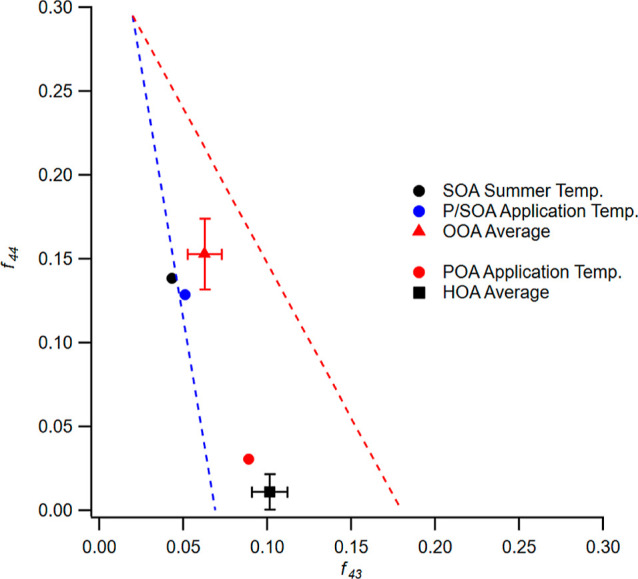
*f*_44_ vs *f*_43_ plot for OA formed under summer and application temperatures (circles) vs averages of four ambient studies of OOA (diamond) and HOA (square) factors. The OOA and HOA averages are bound by 1σ bars. The extent of oxidation was similar for SOA under both temperature regimes and was also similar to that of the ambient OOA factors. By contrast, POA formed under application temperature was less oxidized and was similar to HOA.

We can use the total fraction of AMS organic signals at *m*/*z* = 43 (*f*_43_) and *m*/*z* = 44 (*f*_44_) to characterize the relative oxidation level,^22^ as generally *f*_43_ will decrease and *f*_44_ will increase with oxidation.^23^ In Figure 4, we plot these values for the POA and SOA generated in comparison to HOA and semivolatile OOA factors determined from factor analysis of ambient OA measurements. Unsurprisingly, SOA for both regimes was far more oxidized than POA at application temperature. The average oxidation levels for the HOA and OOA factors were similar to those for POA and SOA, respectively.

To help quantify the similarities of asphalt OA and various HOA, OOA, and diesel emission OA, we compared our asphalt OA spectra to those in the literature. To do so, we collected spectra from the AMS Spectral Database^24^ and compared them using vector angle analysis (see Supporting Information Section S2 for further details). We used diesel bus exhaust^25^ and diesel truck exhaust^26^ as well as HOA and OOA measurements for four different cities.^22,25−27^ For the ambient OOA, two factors were compared from each work.^24,27−29^

Building on the similar extent of oxidation shown above, we can further compare asphalt-related POA and SOA to the ambient HOA and OOA factors using vector angle analysis (Figure S2 in the Supporting Information). For this work, we used two thresholds defined by Moorthy and Sisco: cos θ = 0.7 (θ ≈ 46°) and cos θ = 0.88 (θ ≈ 28°) to mark the lower bound of similarity and strong similarity, respectively.^30^

POA formed from asphalt at application temperatures demonstrated very strong cosine similarities (θ = 12–23°) to all four measured ambient HOA factors. In addition, emissions measurements from the diesel truck and bus exhaust are also similar to the asphalt POA (θ = 13 and 11°, respectively), which is unsurprising as the HOA factors likely encompass diesel and gasoline emissions.^24,26^ This suggests that, in addition to traditional mobile sources such as diesel, asphalt pavements may be an overlooked contributor to HOA in urban environments.

SOA generated from the oxidation of heated asphalt emissions, in contrast, shows a modest similarity to ambient OOA factors (θ = 34–46°). OOA factors associated with semi-volatile (θ = 34–40°) rather than low volatility species (θ = 35–46°) show greater correlation, in line with the volatility of the asphalt OA. This SOA and OOA factor similarity is not nearly as strong as that for asphalt POA and the ambient HOA factor. This is likely due to the enormous, established complexity of atmospheric oxidation products and conditions and thus the resulting OOA. Nonetheless, the modest similarity, together with the comparable extent of oxidation, suggests that the oxidation of heated asphalt emissions is potentially one of many contributors to the OOA in urban environments, especially with regard to semi-volatile fractions.

**Figure 5:**
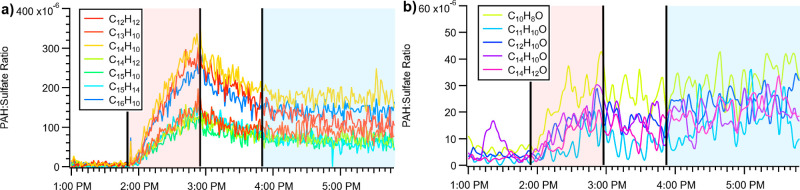
PAH/Sulfate ratio versus time of non-oxygenated (a) and oxygenated (b) PAH species for application temperature emissions, measured with an AMS. The region in red is when heated asphalt vapor injection occurs, and the region in blue is when oxidation occurs. The PAH/sulfate ratio increases during injection, then drops after injection in both cases. During oxidation, the ratio stabilizes for non-oxygenated PAHs and slightly increases for oxygenated PAHs.

An evident component of the C_*x*_H_*y*_ and C_*x*_H_*y*_O_*z*_ at higher *m*/*z* in both temperature regimes was PAH-related, which, alongside other larger aromatic species, are resistant to fragmentation in the AMS, allowing us to measure them directly.^8,14^ PAHs (with and without alkyl substituents) can potentially include a wide variety of hydrocarbon (i.e., C_*x*_H_*y*_) species, as well as oxygenated PAHs (demonstrated in Figure 5b), nitrated PAHs (from the presence of NO_*x*_), hydroxylated PAHs (from – OH addition),^31^ heterocyclic compounds (from heteroatom addition, particularly oxygen), carboxylic acids (from oxidation), aldehydes and ketones,^32^ and PAH quinones.^33^ The most common non-oxygenated PAH ion was C_14_H_10_ (0.07% POA and 0.12% SOA), and the most common oxygenated PAH was C_10_H_8_O (7.1 × 10^–3^% POA and 0.024% SOA). A full list of PAHs measured is included in the Supporting Information (Section S6). In total, observable PAHs account for 0.4% of POA formation and 0.6% of SOA formation, though this is potentially a lower limit given that some fraction of oxidized molecules would partly fragment.

Figure 5a shows the ratio of non-oxygenated PAH ions to sulfate, and Figure 5b shows the ratios for oxygenated PAH ions; specific PAH ions are shown in the aerosol mass spectra in Figure 3. For non-oxygenated and oxygenated PAH species, the PAH/sulfate ratio increases during asphalt heating and subsequently decreases once heating stops. This indicates the evaporation of semi-volatile PAHs from the asphalt, followed by vapor losses to the walls. The PAHs demonstrate a similar semi-volatile nature to other POA, with wall loss rates indicating saturation concentrations of the particulate phase of c* = 0.85 to 0.30 μg m^–3^. Once oxidation starts, the PAH/sulfate ratio increases for oxygenated PAHs due to the oxygenation of other PAH species. Additionally, the PAH/sulfate ratio for nonoxygenated PAHs stops falling and stabilizes. This is likely due to the condensation of oxidation products, retaining aromatic rings.

Hence, the increase in the PAH/sulfate ratio from asphalt heating indicates the release and condensation of PAHs from asphalt. This is especially interesting both due to the health implications of PAH exposure from asphalt as well as its demonstration that relatively high molecular weight species (>100 g/mol) and aromatic compounds readily off-gas from asphalt after heating.

### 3.4 Organic Aerosol Activity Factors

Using the measured organic aerosol and accounting for the steady change in organic/sulfate, we can separate POA emissions and SOA formation from one another in the application temperature experiment,^17^ reporting each as “activity factors.” These activity factors are analogous to fuel-based emission factors commonly used in emissions inventories and have a mass of OA formed per mass of asphalt heated per time (where time refers to the heating duration and thus the total emissions, not the duration of oxidation). Hence, the activity factors have units of mg-(P/S)OA kg-asphalt^–1^ h^–1^. To obtain factors for POA and SOA, we compare measured OA to the amount of asphalt bitumen binder rather than the total mass of asphalt with aggregates, as binder typically composes only ∼5% of total asphalt mass.^7,34,35^ We can calculate our activity factors using the formula: , where *a*_OA_ is our activity factor (mg-OA kg^–1^ asphalt binder heated h^–1^), *V*_C_ is the chamber volume (10 m^3^), Δ*C*_OA_ is the wall-loss corrected production of OA (μg m^–3^), *m*_asp_ is the asphalt mass (g), *X*_binder_ is the mass fraction of asphalt that is binder (0.05), and *t*_heat_ is the time of heating (h). We do not consider the wall loss of SOA precursor vapors before the oxidation starts. Thus, the experiments likely underestimated the SOA production and activity factors.

At summertime heating conditions, we conducted replicate experiments, heating large quantities of asphalt for *t*_heat_ = 3 h, using *m*_asp_ = 109 and 101 g (5.5 and 5.0 g of binder), and formed no POA in addition to SOA formation of Δ*C*_OA_ = 1.10 μg m^–3^ and 1.15 μg m^–3^, respectively, in our *V*_C_ = 10 m^3^ chamber. The SOA activity factors were 0.67 and 0.76 mg-OA kg^–1^ h^–1^, respectively, for an average of 0.72 mg-OA kg^–1^ h^–1^. To directly compare application and summertime heating conditions, we conducted summer temperature experiments with ∼40 g heated for 1–1.5 h and observed no POA or SOA. At application temperature, as shown in Figure 1a, we heated *m*_asp_ = 41.8 g of asphalt (2.1 g of binder) for *t*_heat_ = 1 h and observed POA of Δ*C*_OA_ = 30 and 3.2 μg m^–3^ of SOA. This SOA was determined by taking an exponential fit of the POA concentration pre-oxidation to estimate the remaining POA after wall losses and subtracting from the total organic concentration to get SOA. Here, the POA and SOA activity factors were 142 and 15.3 mg-OA kg^–1^ h^–1^, respectively.

Using these emission factors, our goal is to estimate the OA that could typically be observed in an urban area from these two related sources–street paving and continuous volatilization from existing roadways over the lifetime of the pavement. The emissions at paving temperature are more intense, but the streets are only paved infrequently, and off-gassing from pavement has been shown to persist for more than a decade.^11,12^ It is thus not immediately obvious which contribution will outweigh the other.

We can estimate OA produced during the day of application by assuming that emissions and production occur at a constant rate over an 8 h day, though we acknowledge that the duration of paving and associated/subsequent emissions vary considerably between projects, in addition to variations between asphalt binder composition that can influence emissions.^7^ We also assume that subsequent SOA formation from older roadway surfaces occurs for 8 h during any sufficiently hot and sunny summer day, regardless of pavement age. Our assumption regarding road surface temperature is supported by Chestovich et al. and Li et al., who measured ambient summertime road surface temperatures in Nevada and central California, respectively, reaching or surpassing 55 °C on average 8 h daily.^36,37^ Chestovich’s measurements reached a peak temperature above 70 °C for roughly 4 h daily, suggesting our constant emission factor may be an underestimate.^36^ We therefore estimate application temperature POA and SOA activity factors of 1140 and 123 mg-OA kg^–1^ day^–1^ and a solar heating SOA activity factor of 5.7 mg-OA kg^–1^ day^–1^. This indicates that asphalt loses a considerable amount of immediate OA production potential shortly after asphalt is laid but nonetheless still produces a considerable amount of SOA when heated to typical summertime temperatures.

Although the SOA activity factor for the solar heating condition is considerably lower than the activity factors for application conditions, it has a far greater potential cumulative impact on OA production because SOA will form on all warm summer days, whereas asphalt paving is infrequent (on the order of longer than a decade between paving). Hence, though the immediate activity factors for fresh asphalt are higher, there may be 100s or more days depending on the location of warm surface SOA formation for each day of application. We also note that the solar heating experiments here are isolated on the effect of enhanced temperature and do not include solar irradiation, which will likely increase emission rates.^7^

Here, we use data from Pittsburgh, PA (USA) to estimate the relative contribution of application POA (and SOA) versus SOA formed from rewarmed hot asphalt on warm summer days. GIS (Geographic Information System) shapefile data indicate that Pittsburgh has 944 total miles of roads, of which 216 miles are classified as major roads. The City of Pittsburgh Department of Mobility and Infrastructure paved an average of 47.3 miles of road for 2018–2021 (excluding 2020, when paving was hindered by the COVID-19 outbreak), indicating that approximately 5% of roads are paved annually (https://pittsburghpa.gov/domi/street-resurfacing). Hence, laid asphalt has an approximate lifetime of 20 years.

We then estimate the number of sufficiently hot days using hourly ambient temperature data from the National Oceanic and Atmospheric Administration (https://www.ncei.noaa.gov/). Asphalt reaches considerably hotter temperatures than ambient during hot weather, contributing to the Urban Heat Island effect and enabling SOA formation.^7,38^ We find a mean of 807 h annually between 2006 and 2020, when the ambient temperature in Pittsburgh reached at least 24 °C. This temperature is consistent with an asphalt temperature of 55 °C given an estimated increase in pavement temperature of 30 °C above surface temperature, as noted by Li et al.^37^ Hence, post-application, the summer activity factor will apply for approximately 100 8 h day equivalents annually. It is of course an oversimplification to assume a stepwise change in emissions from zero below 24 °C to “solar heating” above that threshold (noting Lasne et al. observed substantial VOC emissions at lower temperatures);^12^ our purpose is merely to test whether this source should receive further attention. In contrast, a typical application will only occur once every 20 years, and assuming prompt, high emissions persist for 8 h is less extreme.

**Figure 6:**
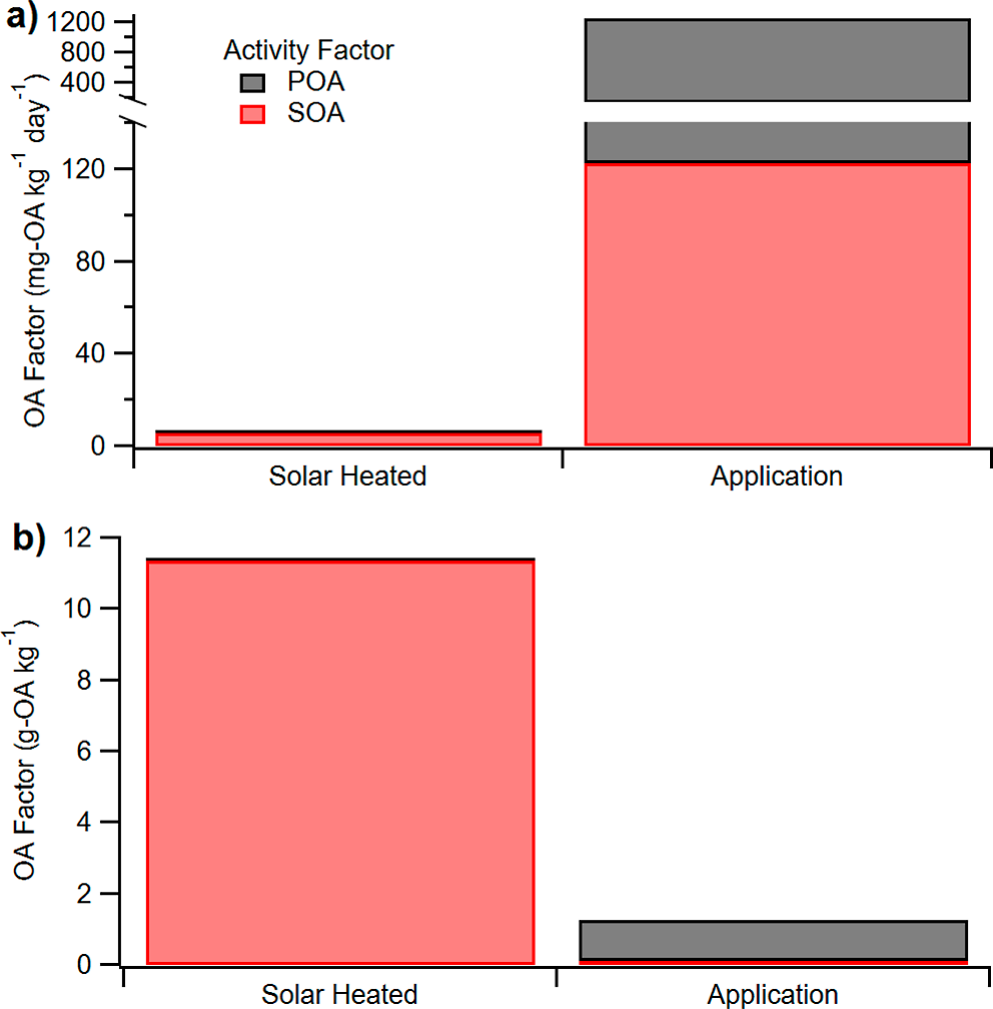
Activity factor in mg-OA/kg per day (8 h) (a) under solar heating (left) and application (right) conditions and lifetime activity factor in g OA/kg binder (b) under solar heated (left) and application (right) conditions. SOA is formed under solar heating conditions at a ratio of ∼1:20 compared to SOA under application conditions. POA substantially outweighed SOA under application temperature. The lifetime activity factors (b) are adjusted for a 20 year lifespan for asphalt before repaving. Despite the lack of significant POA formation and a lower SOA factor at solar heating conditions compared to application, passive SOA from asphalt heating over time has the capacity to outweigh organic emissions from the application process itself.

Figure 6b shows the estimate of total OA production from asphalt over a 20 year roadway lifetime. While POA emissions and subsequent SOA formation on the day of paving are large, this event occurs approximately once every 20 years. In contrast, the low-level SOA-forming emissions from solar heating persist for many years. The result is that total OA production from summertime heating of asphalt surfaces dominates over the high emissions on the day of paving, with the total contribution over 20 years from asphalt application being 1.1 g of POA kg^–1^ binder and 0.1 g of SOA kg^–1^ binder compared to 11.4 g of SOA kg^–1^ binder from passive asphalt heating. Hence, over the asphalt lifetime, solar heating could contribute nearly ten times as much OA as application.

It is important to note that here we assume that the solar-heated SOA precursor emissions that we measure will continue at a constant rate for decades. While recent published results show that heated asphalt emits for more than a decade after initial application,^11,12^ trends in emissions over long time scales with a range of real-word specimens remain uncertain, with recent work showing similar VOC emission rates from real-world aged samples.^12^ We therefore performed additional checks to evaluate the potential for long-term contributions of paved roads to SOA. First, we performed a mass balance on the bitumen binder. Over 20 years, our results suggest the formation of about 11.4 g SOA kg^–1^ binder (1.14% of the total binder); if these binder vapors have high SOA mass yields, consistent with IVOCs,^39^ the total loss from evaporation of the asphalt binder should not be substantially greater, leading to a total of a few percent binder loss to evaporation over the lifetime of the road surface.

Second, in Section S5 of the Supporting Information, we examined the cumulative impact of application temperature P/SOA versus passive heating SOA over the lifetime of a roadway. Figure S5 shows that the cumulative SOA formation from summertime roadway heating equals the application temperature P/SOA emission and production after 2.25 years. Thus, while there remains uncertainty in the long-term emission rates of SOA precursors from passive roadway heating, our SOA production estimates suggest that SOA production from passive heating starts to exceed the lifetime OA impact of asphalt pavements relatively early in the lifetime of the road.

By using these P/SOA factors in addition to road measurements, we can estimate the annual contribution of both asphalt application as well as post-application oxidation in the city of Pittsburgh. The Allegheny County Code in Division 7, Article V, notes the width of streets, with an average of 12 and 24 feet for nonmajor and major roads, respectively. Taken with the road lengths of 944 total miles of roads and 216 miles of major roads from GIS (∼1520 and ∼348 km, respectively), this gives approximately 1.4 × 10^7^ m^2^ of asphalt surface in the city of Pittsburgh. Assuming that all the asphalt binder can contribute to summertime temperature off-gassing emissions over the 20 year pavement lifetimes (rather than only a thin surface layer), we can estimate the asphalt thickness and density as 4.35 cm and 1000 kg m^–3^, respectively, giving a total volume of asphalt of 6.0 × 10^5^ m^3^ and a total mass of asphalt of 6.0 × 10^8^ kg.^7^ Multiplying by 0.05 for the 5% fraction of binder, the total binder mass is thus 3.0 × 10^7^ kg. Hence, multiplying by the annual activity factors, asphalt in Pittsburgh could contribute 1.7 × 10^10^ mg-OA yr^–1^ of SOA from postapplication solar heating of existing roadways, 1.8 × 10^8^ mg-OA yr^–1^ of SOA from asphalt application, and 1.7 × 10^9^ mg-OA yr^–1^ of POA from asphalt application. The corresponding daily summertime amounts are 4.7 × 10^7^ mg-OA day^–1^ of SOA from postapplication solar heating of existing roadways, 5.0 × 10^5^ mg-OA day^–1^ of SOA from asphalt application, and 4.6 × 10^6^ mg-OA day^–1^ of POA from asphalt application.

Pittsburgh is spread over 150 km^2^, meaning that with an estimated boundary layer of 2 km (during hot summer days), the total volume of air over the city is roughly 300 km^3^. Dividing our daily OA formation by this volume, we obtain an annual average daily contribution of 0.16 μg m^–3^ for SOA from post-application asphalt, 0.015 μg m^–3^ for application asphalt POA, and 0.002 μg m^–3^ for application asphalt SOA over Pittsburgh. Hence, post-application asphalt has potential as an SOA contributor, eclipsing the initial application itself. Given that roughly 100 days/year qualify as “hot”, on those days we estimate roughly 0.56 μg m^–3^ of ambient SOA formed from vapors emitted by old, hot roadways, making it a nontrivial source that warrants further consideration.

While this study demonstrates that asphalt emissions can be a significant contributor to urban SOA, there are several limitations, and our results indicate the need for future research. We tested road paving asphalt in one city. Asphalt formulations differ based on end use (e.g., road paving versus roofing); there may be additional differences in formulation based on the location and time of year. In addition, the environmental conditions paved asphalt is exposed to vary dramatically by topography and meteorology, with areas with greater sunshine hours and temperatures likely to be more susceptible to OA formation. The full temperature and time (aging) dependence of SOA precursor emissions require further research, as this source may be significant. Better knowledge of OA formation by asphalt may account for this source in inventories and chemical transport models.

## Supporting Information Available

Description of the calibration gas used for the PTR-MS, cosine similarities, mass spectral differences, saturation concentration calculations, activity factor estimates, and list of PAH species measured (PDF)
