# Supplementary material for: Primary and Secondary Organic Aerosol Formation from Asphalt Pavements
Source: Environ Sci Technol. 2023 Nov 6;57(48):20034–42. doi: 10.1021/acs.est.3c06037 (PMC10702534; doi:10.1021/acs.est.3c06037)
Supplement: Supplementary file 1 — es3c06037_si_001.pdf [file es3c06037_si_001.pdf]

Supporting Information for Primary and Secondary Organic Aerosol Formation from Asphalt Pavements

Mackenzie B. Humes[1], Jo E. Machesky[2], Sunhye Kim[3], Oladayo J. Oladeji[3], Drew R. Gentner[2], Neil M. Donahue[1,4], Albert A. Presto[3]*

1-Department of Chemical Engineering, Carnegie Mellon University, Pittsburgh, Pennsylvania 15213, United States

2-Department of Chemical & Environmental Engineering, Yale University, New Haven, Connecticut 06511, United States

3-Department of Mechanical Engineering, Carnegie Mellon University, Pittsburgh, Pennsylvania 15213, United States

4-Department of Chemistry, Carnegie Mellon University, Pittsburgh, Pennsylvania 15213, United States

*E-mail: apresto@andrew.cmu.edu

Section S.1-Calibration Gas Standard for PTRMS Calibration (S2)
Table S.1- PTRMS calibration gas standard and concentration used during PTRMS calibration. (S2)

Section S.2-Cosine Similarity (S3-S5)
Figure S.2- Similarity angles for asphalt POA & SOA vs HOA & OOA factors (S3)

Section S.3- Mass Spectral Differences (S6)
Figure S.3- Differences in mass spectra fractions between post-oxidation and pre-oxidation for application conditions (S6)

Section S.4- Saturation Concentration (C*) Calculation (S7-S9)
Table S.4a- Select organic, sulfate, and organic loss concentration data from high temperature asphalt heating (S7)

Figure S.4b- Total organic loss vs time for POA before oxidation (S8)

Section S.5- Activity Factor Estimates Over Long Timescales (S10)
Figure S.5- Organic aerosol mass per mass asphalt binder vs time (S10)

Section S.6- Polycyclic Aromatic Hydrocarbon List (S11)
Figure S.6- Fractions of PAH species measured in asphalt POA/SOA (S11)
Section S.7- References (S12)

Section S.1- Calibration Gas Standard for PTRMS Calibration

**Species**	**Mass (g/mol)**	**Calibration Gas Standard (ppb)**	**PTRMS Calibration Concentration (ppb)**
Methanol	32.026215	2070	36
Acetonitrile	41.026549	2060	36
Acetone	58.041865	2070	36
Vinyl Chloride	61.992328	2040	35
Isoprene	68.0626	2050	36
Methyl Vinyl Ketone	70.041865	2090	36
2-Butanone	72.057515	2120	37
Benzene	78.04695	2070	36
Toluene	92.0626	2060	36
1,2-Dichloroethylene	96.960632	2080	36
Styrene	104.0626	2070	36
p-xylene	106.07825	2060	36
1,3,5-Trimethylbenzene	120.0939	2060	36
Trichloroethylene	129.914383	2060	36
1,2,3,5 Tetramethylbenzene	134.10955	2070	36
Tetrachloroethylene	165.872461	2070	36

Table S.1: PTRMS calibration gas standard and concentration used during PTRMS calibration.

Section S.2- Cosine Similarity

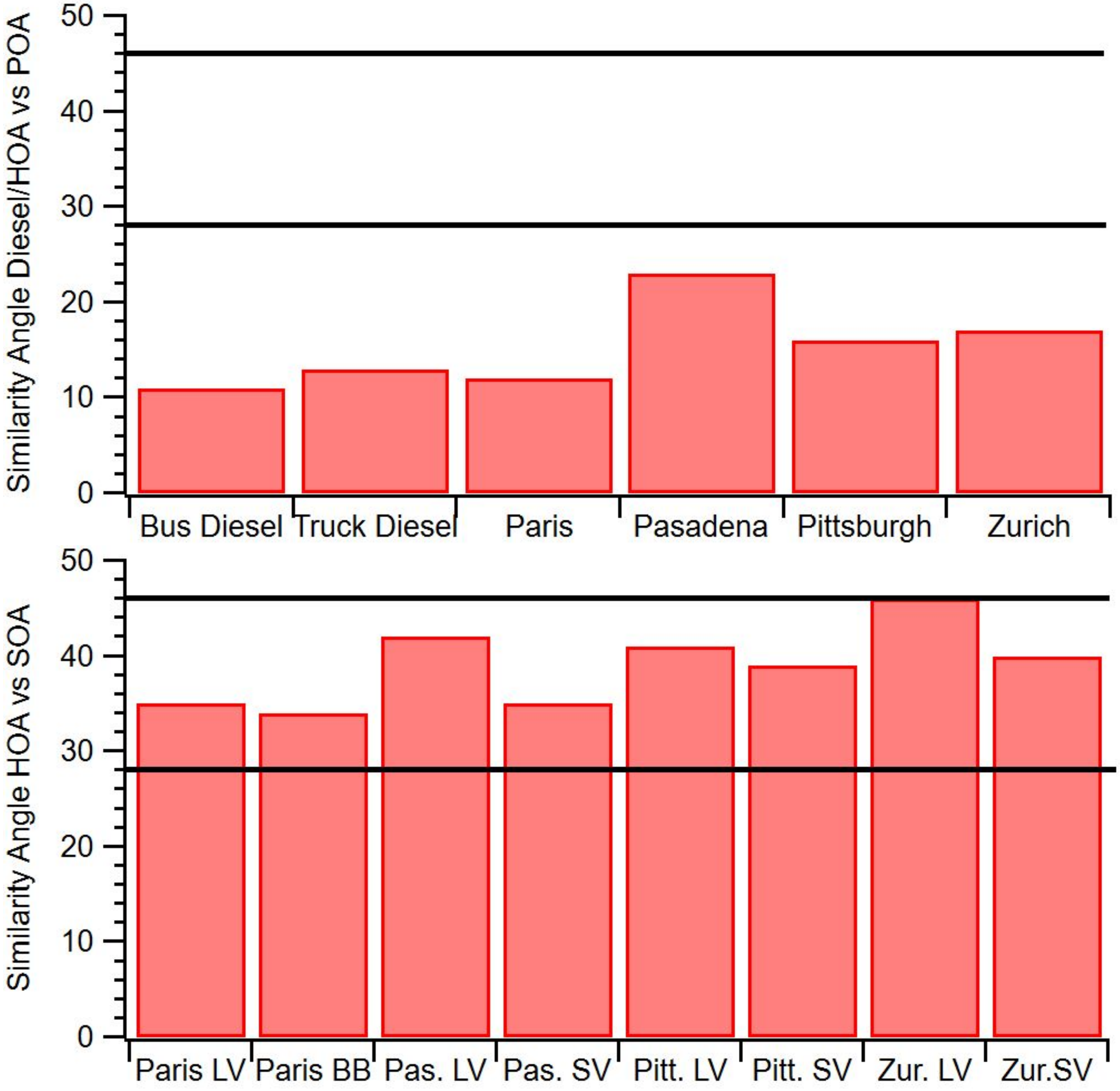

Figure S.2: Vector angle analysis chart for POA vs ambient diesel and HOA factors (above) and SOA vs ambient OOA factors (below). Lines at $\theta = 28°$ and $46°$ represent the minimum thresholds for strong correlation and partial correlation respectively. Asphalt SOA shows partial correlation to various OOA factors ($28° \leq \theta \leq 46°$) with semi-volatile factors having higher correlation than low-volatility factors. Asphalt POA by contrast demonstrated strong correlation ($\theta \leq 28°$ in all cases, and $10° \leq \theta \leq 20°$ except for Pasadena HOA) to HOA factors and diesel emissions.

Sources:

All data were obtained from the AMS Spectral Database (1). For diesel emission OA, we used papers by Canagaratna et al. for diesel bus exhaust (2) and Mohr et al. for diesel truck exhaust (3). For comparison to ambient HOA and OOA, we used papers by Hersey et al. for Pasadena (4), Ulbrich et al. for Pittsburgh (1), Lanz et al. for Zurich (5), and Crippa et al. for Winter in Paris (6). For the ambient OOA, two factors were compared from each work, including semi- and low-volatility OOA in Paris, as well as one low volatility OOA factor (Type 1/LV) and semi-volatile OOA factor (Type 2/SV) for the remaining three cities (1; 4; 5; 6).

Further Information:

In general, HOA factor and vehicle emissions do not vary much, as shown in Figure S.2. Our asphalt POA are very similar to HOA (Figure S.2) and fall into similar extents of oxidation (Figure 4). The similarity is so strong (θ = 11°-23°) that even under considerably different environmental conditions the asphalt POA will still be strongly similar to the HOA factors and vehicle emissions.

OOA factors cover a wide variety of species and can be produced under several different conditions from various sources, which is why we chose to compare with four different cities with dramatically different environments. The differences in environmental conditions may play a part in the similarity analysis as these factors can affect SOA formation and chemistry. For example, higher NO_x levels lead to higher organic nitrate formation. Different formulations of asphalt can affect OA formation as well. Hence, there will certainly be differences in environmental conditions, which form different products, and therefore different similarities to our results.

The AMS uses electron bombardment, which is a hard ionization technique, leading to fragmentation. Hence, similarities in fragmentation from these sources point to similarities in composition, with the total fraction at *m/z* 44 being a particularly important point of comparison, especially with regard to extent of oxidation. Considering this, we see statistical significance in similarity for asphalt SOA with four dramatically different environments and similar fractions at *m/z* 44 (Figures 4 and S.2). This points to asphalt heating as a contributor to OOA in urban environments, especially with regard to semi-volatile fractions. While this work is not an exact, precise measurement of how asphalt should act in an actual urban environment, it does demonstrate that asphalt POA and SOA have statistically significant similarities to HOA and OOA factors respectively across a wide range of environmental conditions.

Section S.3- Mass Spectral Differences

The POA mass spectra can be further compared to spectra post-oxidation by taking the difference in the fragments between them. High NO_x oxidation leads to a dramatic change in the composition of the OA. While C_xH_y still composes the majority of signal, its total drops to 58%, oxygenated species increase to 36%, and NO family ions to 2.5%. This is clear in Figure S.3, where the contribution of C_xH_y fragments drops across the mass range, again with 14 *m/z* features from adding a single carbon to the backbone. The majority of species increasing in fraction are oxygenated hydrocarbons and NO_x, the latter indicating substantial organic nitrate formation. Two especially important changes for fragments are the increase in CO_2^+ and the decrease in $C_3H_7^+$, which indicates strong oxidation. This extent of oxidation, as well as the overall mass spectra plays a major role in comparing asphalt OA to emissions factors.

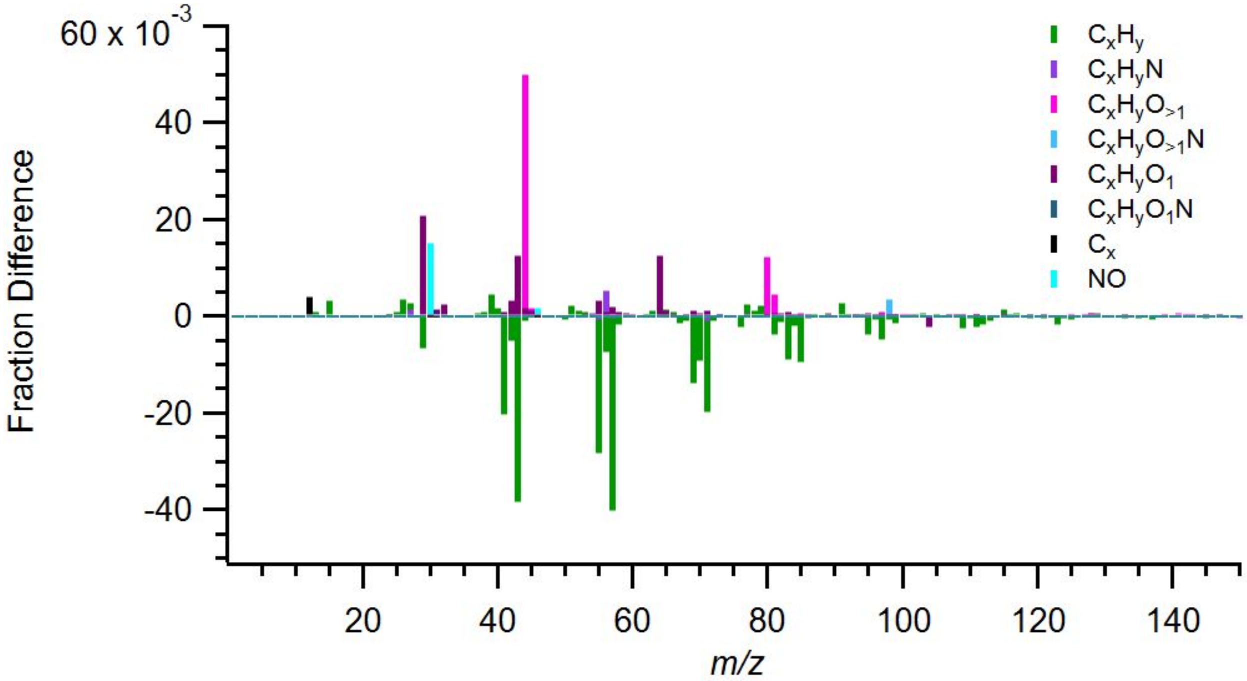

Figure S.3: Differences in mass spectra fractions between post-oxidation and pre-oxidation for application conditions colored by hydrocarbon family. Positive numbers indicate species found in greater abundance post-oxidation while negative numbers indicate species more common pre-oxidation (POA). The graph shows a dramatic increase in the fraction of oxygenated and nitrogenated species post-oxidation.

Section S.4- Saturation Concentration (c^*) Calculation

The mean saturation concentration (c^*) is a material property based on the chemical structure that would not change based on the mass loading. c^* is calculated as $c^* = \frac{M_i \xi_i p_{L,i}^0}{760RT}$, where M_i is the molecular weight, ξ_i is the activity coefficient (typically assumed to be 1), p_L^0 is the saturation vapor pressure of the pure compound, R is the gas constant, and T is the temperature. Changes in the oxidation environment may lead to different compound formation, which would change individual c^*. In our case, we measure based on the entire organic species using wall loss to find the volatility and therefore c^*.

The saturation concentration is calculated in a series of steps. First, we calculate the loss rate for each time interval using the equation: $l_{org}^{W}[\mu g\ m^3\ s^{-1}] = -\frac{\partial(C_{org}^P : C_{sulf}^P)}{\partial t}\frac{C_{sulf}^P}{CE}$, where C^P_{org}:C^P_{sulf} is the organic:sulfate ratio, C^P_{sulf} is the concentration of sulfate, and CE is the collection efficiency of the AMS, which we can estimate at ~0.2 for ammonium sulfate seed particles.

For example, let us look at three different sequential times from the high temperature asphalt heating:

**Time**	**Organic ($\mu g\ m^{-3}$)**	**Sulfate ($\mu g\ m^{-3}$)**	**Organic:Sulfate**	**lw_org ($\mu g\ m^{-3}\ min^{-1}$)**	**Lw_org ($\mu g\ m^{-3}$)**	**Sum_Lw ($\mu g\ m^{-3}$)**
15:08	25.28	73.95	0.3419	-	-	-
15:09	24.61	72.04	0.3416	0.1138	0.1138	0.1138
15:10	24.06	70.45	0.3415	0.0198	0.0198	0.1336
15:11	24.01	71.13	0.3375	1.410	1.410	1.543

15:12	23.66	70.81	0.3342	1.188	1.188	2.731

Table S.4a: Select organic, sulfate, and organic loss concentration data from high temperature asphalt heating.

At time 15:09 the concentration of organics and sulfate are 24.6 μg m^{-3} and 72.0 μg m^{-3} respectively, giving an organic to sulfate ratio of ~0.3416. At time 15:08, the concentrations of organics and sulfate are 25.3 μg m^{-3} and 74.0 μg m^{-3} respectively, giving an organic to sulfate ratio of ~0.3419. From 15:08 to 15:09 we get an estimated loss difference of: $l_{org}^{W} = -\frac{\partial\left(C_{org}^{P}:C_{sulf}^{P}\right)}{\partial t}\frac{C_{sulf}^{P}}{CE} = -\frac{(72.0\,\mu g\,m^{-3})(0.3416 - 0.3419)}{(1min)(0.2)}$ = 0.11 μg m^{-3} min^{-1}. With a time interval of 1 minute between both, this means a loss of 0.11 μg m^{-3} for this time period. We can repeat this at time 15:10 to get a loss of ~0.02 μg m^{-3} and add at each step to obtain a sum total of 0.13 μg m^{-3}.

The next step is to graph the total mass loss of organics vs time. Over the POA loss range before oxidation, we can fit:

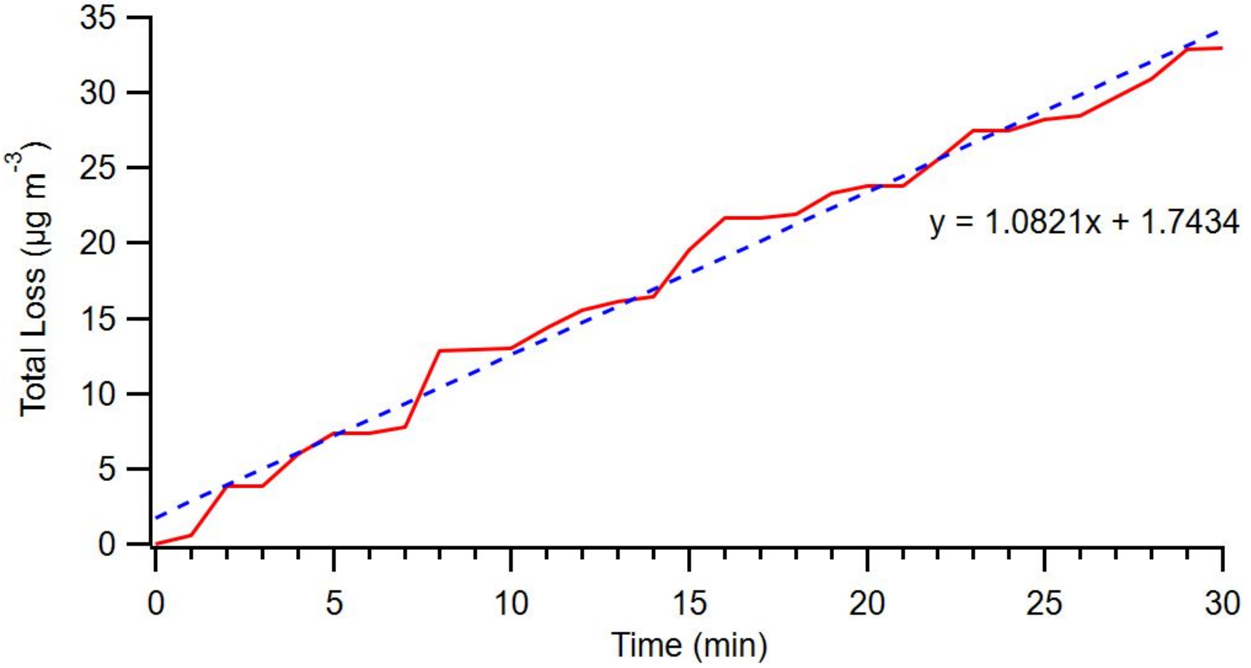

Figure S.4b: Graph of total organic loss vs time for POA before oxidation. The trend line fit to the data is used to estimate the rate of organic loss, which can be used to find the saturation concentration.

The final step is to divide the loss rate by the condensation sink as per the equation: l_{org}^{W} $[\mu g\, m^{3}\, s^{-1}] = a_{org}^{P} C_{org}^{*} \frac{1}{\frac{1}{CS^{P}} + \frac{1}{CS^{W}}} \approx CS^{W} a_{org}^{P} C_{org}^{*}$, where the activity, a, equals 1 and the condensation sink is 0.065 min^{-1}. Hence, $c° = \frac{L_{org}^{W}}{CS^{W}} = \frac{1.08\, \mu g\, m^{-3}\, min^{-1}}{0.065\, min^{-1}} =$ 16 µg m^{-3} for high temperature asphalt POA.

84 Section S.5- Activity Factor Estimates Over Long Timescales

We assume that the SOA formation potential of roadways heated to 55° C remains constant over the ~20-year lifetime of the roadway. Figure S.5 compares the cumulative impact of SOA+POA from the day of paving to SOA formation from passive roadway heating.

SOA+POA formation from the day of paving is a constant; these emissions and the subsequent SOA formation are assumed to occur only on the day that the road is paved. Thus, the Application Temperature emissions are shown as a horizontal blue line.

The red line shows the cumulative production of SOA from passive roadway heating over time. The impact of passive SOA formation equals the emissions from the day of paving after 2.25 years. Thus, our basic conclusion that roadways can be an important source of urban SOA does not require the assumption of constant emissions over decades.

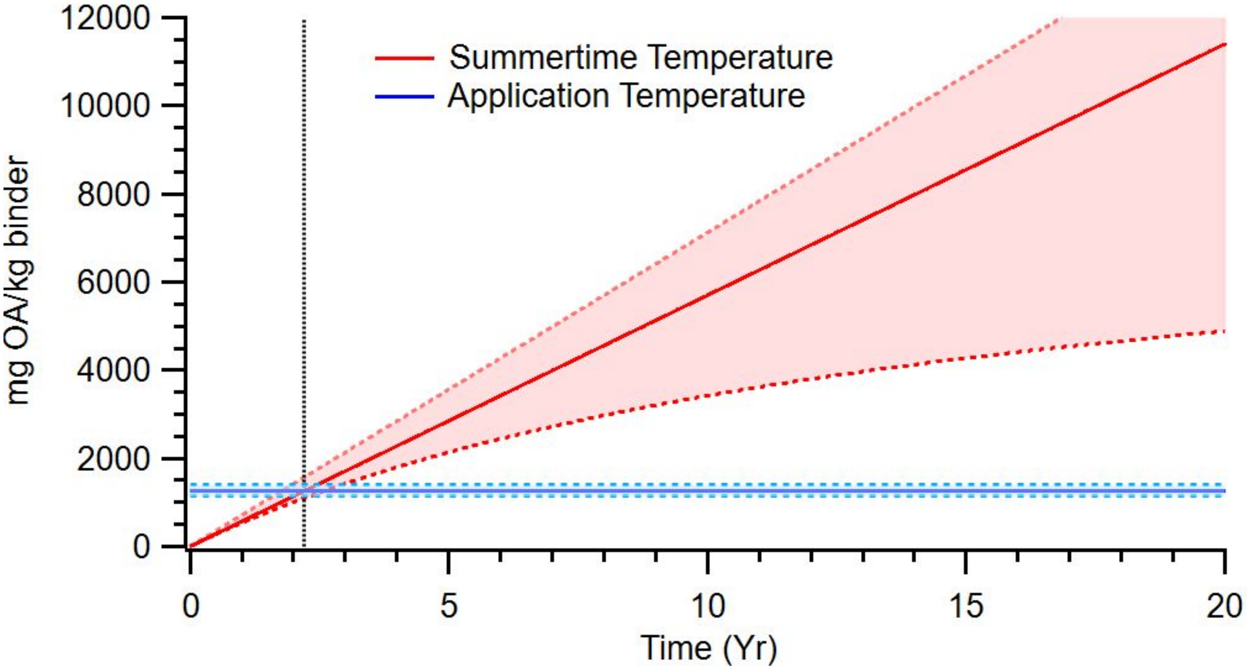

Figure S.5: Organic aerosol mass per mass asphalt binder vs time since paving with ranges marked by striped lines. The bounds for application temperature were marked by 1 standard deviation (σ). The upper bound for summertime temperature was also marked by 1 σ, whereas the lower bound was fit based on a max 1 σ lower than the yield with a more gradual release: $SOA = \frac{yield \times time}{{}^{1}/{}_{1 + yield \times time/\max yield}}$. Within approximately 2.25 years of paving, the total OA emissions from summertime heating overtakes the initial paving at application temperature. Over an approximate asphalt road lifetime of 20 years, the total OA from solar heating will be approximately 10 times greater than application. However, OA formation at summertime temperature has a far greater degree of uncertainty than at application temperature, and this ratio may be greater or smaller.

Section S.6- Polycyclic Aromatic Hydrocarbon List

Oxygenated PAH	POA	SOA
C12H10O	4.24E-05	1.67E-04
C11H10O	4.28E-05	1.48E-04
C14H12O	4.64E-05	1.65E-04
C14H10O	4.69E-05	1.63E-04
C10H8O	7.07E-05	2.36E-04
Non-Oxygenated PAH	POA	SOA
C15H10	2.65E-04	4.52E-04
C14H12	3.00E-04	4.96E-04
C15H14	3.17E-04	3.66E-04
C13H12	3.22E-04	4.91E-04
C13H10	3.42E-04	5.98E-04
C16H10	5.61E-04	9.85E-04
C12H12	6.57E-04	7.81E-04
C14H10	6.98E-04	1.17E-03

Figure S.6: Fractions of PAH species measured in the AMS in asphalt POA and SOA. Non-oxygenated species PAHs are more prevalent than oxygenated PAHs and the total fraction of PAHs is greater in SOA than POA.

Section S.7- References

1. *Interpretation of organic components from Positive Matrix Factorization of aerosol mass spectrometric data.* **Ulbrich, I. M., Canagaratna, M. R., Zhang, Q., Worsnop, D. R., & Jimenez, J. L.** 9, 2009, Atmospheric Chemistry and Physics, Vol. 9, pp. 2891–2918.

2. *Chase studies of particulate emissions from in-use New York City vehicles.* **Canagaratna, M. R., Jayne, J. T., Ghertner, D. A., Herndon, S., Shi, Q., Jimenez, J. L., … Worsnop, D. R.** 6, 2004, Aerosol Science and Technology, Vol. 38, pp. 555–573.

3. *Characterization of primary organic aerosol emissions from meat cooking, trash burning, and motor vehicles with high-resolution aerosol mass spectrometry and comparison with ambient and chamber observations.* **Mohr, C., Huffman, J. A., Cubison, M. J., Aiken, A. C., Docherty, K. S., Kimmel, J. R., … Jimenez, J. L.** 7, 2009, Environmental Science and Technology, Vol. 43, pp. 2443–2449.

4. *The Pasadena Aerosol Characterization Observatory (PACO): Chemical and physical analysis of the Western Los Angeles basin aerosol.* **Hersey, S. P., Craven, J. S., Schilling, K. A., Metcalf, A. R., Sorooshian, A., Chan, M. N., … Seinfeld, J. H.** 15, 2011, Atmospheric Chemistry and Physics, Vol. 11, pp. 7417–7443.

5. *Source apportionment of submicron organic aerosols at an urban site by factor analytical modelling of aerosol mass spectra.* **Lanz, V. A., Alfarra, M. R., Baltensperger, U., Buchmann, B., Hueglin, C., & Prévôt, A. S. H.** 6, 2007, Atmospheric Chemistry and Physics, Vol. 7, pp. 1503–1522.

6. *Wintertime aerosol chemical composition and source apportionment of the organic fraction in the metropolitan area of Paris.* **Crippa, M., Decarlo, P. F., Slowik, J. G., Mohr, C., Heringa, M. F., Chirico, R., … Baltensperger, U.** 2, 2013, Atmospheric Chemistry and Physics, Vol. 13, pp. 961–981.
